# Immunomodulatory Effects of Nanoparticles on Dendritic Cells in a Model of Allergic Contact Dermatitis - Importance of PD-L2 Expression

**DOI:** 10.21203/rs.3.rs-3069059/v1

**Published:** 2023-07-12

**Authors:** Angela Wong Lau, Jessica Perez Pineda, Lisa A. DeLouise

**Affiliations:** 1 Department of Biomedical Engineering, University of Rochester, Rochester, New York, USA.; 2 Department of Dermatology, University of Rochester Medical Center, Rochester, New York, USA.

## Abstract

Nanoparticle (NP) skin exposure is linked to the increased prevalence of allergic contact dermatitis. In prior studies using the mouse contact hypersensitivity (CHS) model, we reported that silica 20 nm (Si20nm) suppressed the allergic response and TiO_2_ doped with manganese (mTiO_2_) exacerbated it. In this work, we conducted *in vitro* experiments using bone marrow-derived dendritic cells (BMDCs) to study the combinatorial effect of the potent 2, 4-dinitrofluorobenzene (DNFB) hapten sensitizer with Si20nm and mTiO_2_ NPs on BMDC cytotoxicity, cytokine secretion and phenotype using the B7 family ligands. Results show that DNFB and mTiO_2_ behave similarly and exhibit proinflammatory characteristics while Si20nm promotes a naive phenotype. We observe that the B7-H3 (CD276) ligand is only expressed on CD80+ (B7–1) BMDC. Results from adoptive transfer CHS studies, combined with BMDC phenotype analysis, point to the importance of PD-L2 expression in modulating the adaptive immune response. This work identifies metrics that can be used to predict the effects of NPs on contact allergy and to guide efforts to engineer cell-based therapies to induce antigen specific immune tolerance.

## INTRODUCTION

The prevalence of allergic skin disorders and adverse skin reactions are on the rise worldwide, contributing to severe morbidity, and having a significant impact on the patient quality of life ^1,2^. It was estimated that the median prevalence of contact allergy is 21.2 % (range 12.5–40.6 %) in the general population of North America and Western Europe^3^. Irritant contact dermatitis results from an acute activation of the innate skin immune system following skin exposure to various types of chemicals (e.g. soaps, perfumes, solvents)^4^. Exposure to low molecular weight haptens can induce an adaptive immune response resulting in allergic contact dermatitis (ACD)^4–7^. In the sensitization phase of ACD, antigen-presenting cells in the skin get activated. They uptake the haptenized proteins and migrate to the skin-draining lymph nodes where they present antigen to naïve T-cells that differentiate into CD8+ and/or CD4+ effector and memory T cells. Noncirculating tissue-resident memory (T_rm_) cells seed the skin in 14–30 days and persist long term (>1 yr) in mouse models of ACD^8,9^ In the challenge phase, re-exposure to the hapten activates the antigen-specific memory T cells to induce an allergic response.

There are numerous chemicals in the home, workplace, and in environment that can contribute to irritant and/or ACD. Recent studies suggest that environmental factors, including nanoparticles (NPs), can influence the prevalence and course of allergic disease, and NPs are suspected to be a contributing factor to the rise of ACD ^10–12^. Sources of NPs are numerous. Natural sources include forest fires, soil erosion, dust storms, and volcanic activity, whereas anthropogenic sources include those produced in the laboratory for commercial use and those present in air pollution from factories, automobiles, and cigarette smoke^13–15^. Particulates in air pollution are known to induce oxidative stress and inflammation in the skin^16,17^. Studies suggest that oxidative stress in the skin is an initiating event that results in an immunosuppressive ACD response by activating the platelet-activating factor receptor (PAF-R) signaling pathway^18,19^. Prior studies in our lab reported that engineered NPs can modulate the adaptive immune response in a mouse model of allergic contact hypersensitivity (CHS)^20^. We observed that small (<200 nm) negatively charged NPs, independent of composition, suppressed the allergic response in the challenge phase but similar sized positive NPs did not^21^. Moreover, certain NPs including highly carboxylated carbon nanotubes (CNT) and some TiO_2_ NPs could exacerbate the allergic response^21–24^. The mechanisms of how NPs alter the adaptive immune response in the skin remain unclear, however, we did observe that NPs modulated early signaling events in the challenge phase (<2 hr)^21^. Since, NPs do not readily diffuse through the skin barrier as small molecular weight chemical haptens^20,25–29^, it seems plausible that the NPs must alter epidermal derived signals that can affect mast cell (MC) and/or dendritic cell (DC) activation or function. These two cell types are critically important in transducing the CHS allergic response^30–32^. Mast cell deficiency in the skin dramatically reduces the allergic response due in part to impaired emigration of skin DCs to the lymph node in the sensitization phase ^32–34^. Activated MC are an important source of TNFα that promote DCs to migrate to lymph nodes^34^. Furthermore, MC and DCs interact to activate each other^7,32^

Recent studies in mice found that skin DCs (CD11c+, MHCII+), specifically epidermal Langerhans cells (CD103−, EpCAM+) and conventional cDC2 (CD103−, CD11c+, CD11b+), acquired OVA antigen in the skin and primed T cells in the lymph node but Langerhans cells were not required or sufficient to induce effector T cell response^35^. In fact, the function of epidermal Langerhans cells in the development of the CHS adaptive immune response has been debated and intensely studied for years using genetic ablation strategies ^36–40^. It is now accepted that the intensity of the CHS response correlates directly with the efficiency of T cell priming, and that LCs are important only at low hapten exposure. Dermal DCs subsets, specifically cDC2, are mainly responsible for the initiation and activation of the CHS response^31,38,41^. While, it is recognized that the physiochemical properties of NPs can alter DC function^42^, how NPs may alter phenotypic changes in cDC2 to alter the efficiency of T cell priming in the context of skin allergy is unknown.

In this work, we focus on examining the effect that NPs have on the phenotype and activation of bone marrow-derived (CD11c+, MHCII+) dendritic cells (BMDC). Specifically, we studied a ~20 nm negatively charged silica NPs (Si20nm) that suppressed the *in vivo* CHS response and a ~50 nm negatively charged manganese-doped TiO_2_ NPs (mTiO_2_) that exacerbated it^21^. Using flow cytometry, cytokine analysis, and the well-established dinitrofluorobenzene (DNFB) CHS mouse model, we characterized the BMDC phenotype focusing on several B7 family of co-stimulatory markers^43^ including CD86 (B7–1), CD80 (B7–2), PD-L1 (B7-H1), PD-L2 (B7-DC) and CD276 (B7-H3).

CD86 and CD80 are classic markers of DC activation and they have dual binding capacity to CD28, an activating T cell receptor that is constitutively expressed on naïve T cells and CTLA- a regulatory receptor which is upregulated upon T cell activation^44^. The dual binding capacity of CD86/CD80 to T cell receptors that enhance (CD28) or suppress (CTLA-4) proliferation has been the subject of much investigation^45–48^. CD80 and CD86 act cooperatively to modulate T-cell activation and tolerance induction^43,47^. It is believed that T cell fate is driven by the relative expression levels of the CD86/CD80 ligands on the DC and the CD28/CTLA-4 T cell receptors^46,49,50^ as well as the fact that the binding affinity of CD86/CD80 ligands to CTLA-4 is ~10x stronger than to CD28 which leads to competitive binding between the activating and regulatory receptors^44,51,52^.

Programmed death ligand 1 (PD-L1) and PD-L2 are ligands expressed on DCs that bind the PD-1 receptor on activated T-cells. Activation of the PD1 receptor inhibits T cell proliferation and proinflammatory cytokine production to suppress immune responses^53^. PD-L1 (B7-H1) is widely expressed on many cell types, including cancer cells, whereas PD-L2 (B7-DC) is only expressed on DCs^52^. It is reported that PD-L2 binds PD-1 with 2 to 6-fold stronger affinity compared with PD-L1^54,55^. Others report that PD-L1 and PD-L2 bind PD-1 with comparable affinities but exhibit striking differences in their PD1 receptor association and dissociation characteristics^56^. Since PD-L1 and PD-L2 expression levels depend on distinct stimuli, it is suggested that they may have overlapping and differential roles in regulating T_H_1 and T_H_2 T cell responses ^57,56^. Studies suggest PD-L2 positive DCs are needed to induce allergen tolerance by generating regulatory T cells (T regs)^58^ and they contribute to generating potent LAP+ Tregs^35^.

CD276 (B7-H3) is a member of the B7 immune checkpoint family and is thought to promote an immunosuppressive response as it is highly expressed in many cancers that correlate with poor clinical outcomes^59^. Our interest in this marker stems from a study that showed activation of the arylhydrocarbon receptor in BMDCs generated Tregs cells that suppressed the allergic CHS response in mice^60^. Analysis of the DC markers (MHCII, CD86, PD-L1, B7-H3, B7-H4) following arylhydrocarbon receptor agonist exposure revealed a marked upregulation of B7-H3 which was associated with the immunosuppression^60^.

In this study we identified key markers of BMDC activation by proinflammatory stimuli (LPS, DNFB, mTiO_2_) including a strong upregulation of the CD86, CD80, and PD-L1 (B7-H1), as well as a weak upregulation of CD276 and a negligible to slight downregulation of PD-L2 (B7-DC). Because of the dual binding affinity of CD80 and CD86 ligands to the CTL-4 (regulatory) and CD28 (activating) T-cell receptors, we further examined how the expression levels of PD-L1, PD-L2 and CD276 differed among the CD80/CD86 subpopulations compared to unstimulated immature BMDCs (imDCs). We observe that CD276 is only expressed on CD80+ cells and that both NPs modulate its expression. In single exposure studies, we find that DNFB and mTiO_2_ behave similarly and exhibit proinflammatory characteristics. In contrast, Si20nm is cytoprotective and promotes a naive imDC phenotype, particularly in DNFB co-culture studies. While PD-L1 is upregulated by proinflammatory stressors, PD-L2 is not and in fact, is down regulated by mTiO_2_ exposure which correlates with an exacerbation of the allergic response in the *in vivo* adoptive transfer contact hypersensitivity (CHS) mouse model. These results show that NPs co-cultured with a potent sensitizer can alter the BMDC phenotype to effect the efficiency of T cell priming and the intensity of the CHS response in both the sensitization and challenge phases. This work points to metrics that can be used to predict the effects of NPs on contact allergy and to the novel use of NPs to engineer immunomodulatory responses in contact allergy.

## RESULTS

### Effects of DNFB, Si20nm and mTiO_2_ on BMDC toxicity, cytokine secretion and co-stimulatory molecule expression

**Figure 1:**
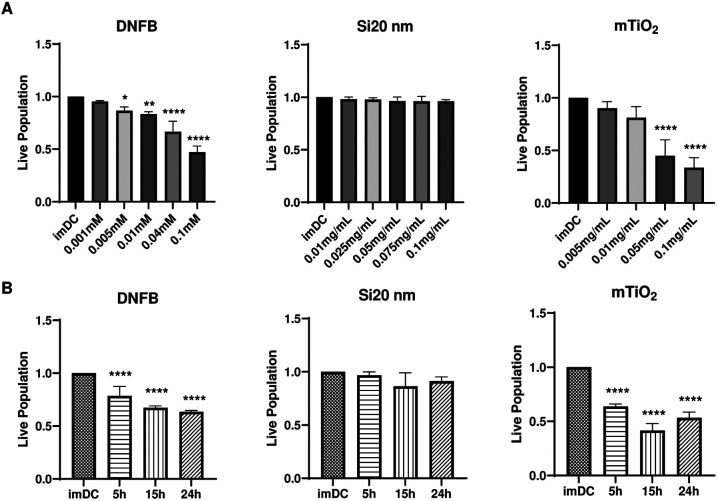
Effects of DNFB, Si20nm and mTiO_2_ exposure on BMDC cytotoxicity as a function of concentration and time. BMDCs were harvested on day 8 and treated with DNFB, Si20nm and mTiO_2_ to study cytotoxicity as a function of concentration and time. (**A**) cell viability as a function of concentration for a 1 hr exposure. (**B**) cell viability following exposure to the lowest non-cytotoxic concentrations from (**A**) and exposed over a period of 5 hr, 15 hr and 24 hr. Cytotoxicity of DNFB and mTiO_2_ NPs on BMDCs was dose- and time- dependent. Live population was normalized against imDC. Ordinary one-way ANOVA was performed and compared to imDC. N=3–5. Mean ± SD. *p < 0.05, **p < 0.01, ***p < 0.001, ****p < 0.0001.

We performed cytotoxicity studies to establish concentration ranges to examine the effects of NPs and DNFB on BMDC phenotypes. We conducted single exposure studies to each stressor for 1 hr to measure cytotoxicity as a function of concentration. Results (Fig. 1A) show a significant dose-dependent decrease in cell viability for DNFB and mTiO_2_ but not for Si20nm. Exposing cells for 1 hr to DNFB (0.1 mM) or mTiO_2_ (0.05 mg/ml) caused a ~50 % decrease in cell viability whereas exposing cells to Si20nm was not cytotoxic. The toxic effects of DNFB correlated with notable changes in the BMDC morphology viewed in bright-field and in TEM images, with cells becoming round and losing dendrites **(Figs. S1-S2**). NP uptake in endosomal vesicles was also confirmed **(Figs. S3-S4**). A non-cytotoxic concentration of each stressor was selected and the BMDCs were exposed for 5 hr, 15 hr, and 24 hr (Fig. 1B). Results again show a significant decrease in cell viability over time for DNFB (0.001 mM) and mTiO_2_ (0.005 mg/ml) but not for Si20nm (0.01 mg/ml).

**Figure 2:**
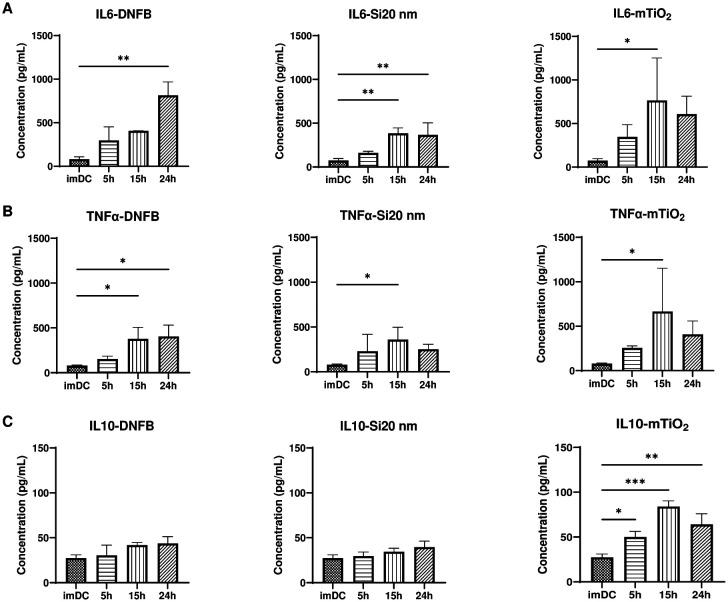
Effects of DNFB, Si20nm and mTiO_2_ exposure on BMDC cytokine secretion. BMDCs were exposed to a low non-cytotoxic concentrations of each stressor (0.001 mM DNFB, 0.01 mg/mL Si20nm and 0.005 mg/mL mTiO_2_) over a period of 5 hr, 15 hr and 24 hr. ELISA was used to analyze cytokines in cell culture supernatants: (**A**) IL-6, (**B**) TNFα, (**C**) IL-10. All stressors tend to increase IL-6 and TNFα. Only mTiO_2_ increased IL-10. Concentration was normalized against % of live cells. Ordinary one-way ANOVA was performed and compared to imDC. N=3–5. Mean ± SD. *p < 0.05, **p < 0.01, ***p < 0.001, ****p < 0.0001.

Cytotoxicity studies suggest that DNFB and mTiO_2_ behave similarly and are likely potent proinflammatory stressors compared to Si20nm. To further characterize interactions with BMDCs, we evaluated the proinflammatory (IL-6, TNFα) and immunosuppressive (IL-10) cytokines secreted in the supernatant by ELISA. BMDCs were exposed to DNFB (0.001 mM), Si20nm (0.01 mg/ml) and TiO_2_ (0.005 mg/ml) as a function of time. Unexpectedly, results showed that each compound increased IL-6 (Fig. 2A) and TNFα (Fig. 2B) secretion and exposure to mTiO_2_ upregulated IL-10 (Fig. 2C). One-hour exposures as a function of concentration also showed that mTiO_2_ rapidly upregulated IL-6, TNFα, and IL-10 (**Fig. S5**). These results suggest that mTiO_2_ is a potent proinflammatory stressor, behaving similarly to LPS (50 ng/ml), that also upregulated IL-6, TNFα and IL-10 over time (**Fig. S6).** These observations were corroborated by intracellular flow cytometry staining for IL-10 and TNFα as a function of concentration and time (**Figs. S7-S8)** but interestingly, in contrast to mTiO_2_, we observe that Si20nm upregulates IL-10 without upregulating TNFα which may suggest an immunosuppressive potential for Si20nm exposure.

**Figure 3:**
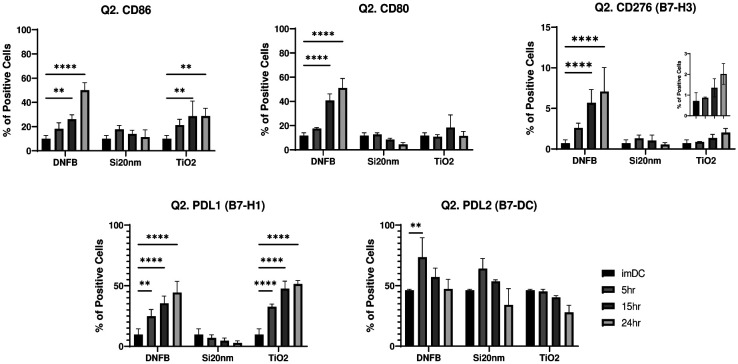
Co-stimulatory molecules from the B7 family quantified by flow cytometry gated on the CD11c+MHCII+ subpopulation following BMDC exposure to each stressor over time. The phenotypic characteristics of BMDCs following exposure to a low concentration of each stressor (0.001 mM DNFB, 0.01 mg/mL Si20nm and 0.005 mg/mL mTiO_2_) was followed over time by flow cytometry gated under the CD11c+MHCII+ (Q2.) subpopulation. DNFB and mTiO_2_ behave more similar with strong upregulation of CD86, CD276, and PD-L1 over time. Only DNFB upregulated CD80. Exposure to Si20nm did not alter these B7 ligands over time. Ordinary one-way ANOVA was performed and compared to imDC. N=3–5. Mean ± SD. *p < 0.05, **p < 0.01, ***p < 0.001, ****p < 0.0001.

Next, we examined the effect of each stressor on the phenotype of the BMDCs by measuring the expression levels of the co-stimulatory molecules by flow cytometry. Single and double gating strategies were used (**Fig. S9)** where single gating assesses the expression levels of each marker on the live cell population, and double gating analyzes the CD11c+MHCII+ subpopulation. To interpret results, we compared them to LPS exposure (50 ng/ml) over time which, for single gating (**Fig. S10)** and double gating (**Fig. S11),** induced the expression of CD86, CD80, and PD-L1. CD276 increased early (3–6 h) and then returned to baseline and no changes in PD-L2 expression were induced by LPS. Exposing BMDC to DNFB (0.001 mM), Si20nm (0.01 mg/ml), and mTiO_2_ (0.005 mg/ml) caused differential changes in the CD11c+MHCII+ phenotype as a function of time (Fig. 3) with DNFB producing effects most similar to LPS exposure. DNFB and mTiO_2_ upregulated CD86 and PD-L1 expression whereas CD80 and CD276 were only upregulated by DNFB over time. PD-L2 was expressed on ~50 % of imDC and exposure to each stressor did not alter its expression. Exposure to Si20nm did not alter any co-stimulatory molecule suggesting BMDC maintained a naïve phenotype. Taken together, the results suggest that DNFB and mTiO_2_ induce an activated BMDC phenotype and that mTiO_2_ may be a more potent stressor as it failed to upregulate CD80, which is important for binding CTLA-4 to promote regulation.

**Figure 4:**
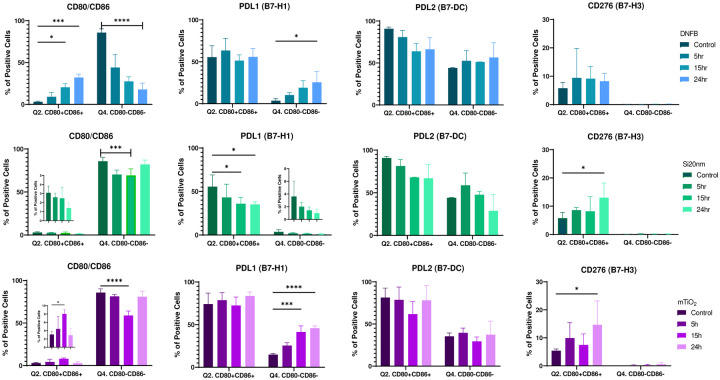
Co-stimulatory molecules from the B7 family quantified by flow cytometry gated on the CD11c+MHCII+ and CD80/CD86 subpopulations following BMDC exposure to each stressor over time. The phenotypic characteristics of BMDCs following exposure to a low concentration of each stressor (0.001 mM DNFB, 0.01 mg/mL Si20nm and 0.005 mg/mL mTiO_2_) was followed over time, 5 hr, 15 hr, and 24 hr, by flow cytometry gated under the CD11c+MHCII+ CD80/CD86 subpopulations. Presented here are changes the CD80+CD86+ double positive (DP) (Q2.) and the CD80-CD86- double negative (DN) (Q4.) subpopulations including the expression levels of PD-L1 (B7-H1), PD-L2 (B7-DC) and CD276 (B7-H3) on the DP and DN subpopulations. For DNFB exposure, the activated DP population clearly increases as the naive DN decreases and PD-L1 expression increased on the DN at 24 hr. mTiO2 exposure over time parallels the effects of DNFB exposure. In contrast, Si20nm exposure over time tended to decrease the DP subpopulation and it significantly decreased PD-L1 expression in the DP subpopulation. Both Si20nm and mTiO_2_ tended to increased CD276 in the DP at 24 hr. The similarities between DNFB and mTiO_2_ were evident in CD80/CD86 and PD-L1 expression and distinct from Si20nm. A two-way ANOVA was performed and compared to imDC. N=3–5. Mean ± SD. *p < 0.05, **p < 0.01, ***p < 0.001, ****p < 0.0001.

The differential effects of each stressor on the expression of CD80 and CD86 led to investigating the expression of PD-L1, PD-L2, and CD276 within the CD86/CD80 subpopulations over time (Fig. 4). Results showed that exposure to DNFB (0.001 mM) decreased the naïve CD80^−^CD86^−^ double negative (DN) population and induced a steady significant rise in the activated CD80^+^CD86^+^ double positive (DP) population which are trends expected for a proinflammatory stressor. Similarly, mTiO_2_ (0.005 mg/ml) showed a trend toward upregulating the DP population, whereas Si20nm (0.01 mg/ml) exposure over time did not. Both NPs tended to downregulate slightly the DN subpopulation up to 15 hr but not as definitively as DNFB. This subtle decrease in the DN is due primarily to the upregulation of CD80+ single-positive cells (**Fig. S12**). The expression levels of CD276, PD-L1 and PD-L2 in the CD80/CD86 subpopulations also differed depending on the stressor with DNFB and mTiO_2_ exhibiting similar trends. Specifically, DNFB and mTiO_2_ exposure up-regulated PD-L1 in the DN (Fig. 4) and the single positive subpopulations (**Fig. S12**) with no change in the DP subpopulations. In contrast, Si20nm exposure down-regulated PD-L1 expression in the DP subpopulation over time with no change in DN subpopulation. No changes in PD-L2 expression in DP or DN subpopulations were induced by either stressor (Fig. 4) but decreases in PD-L2 expression in the CD86 and CD80 single positive cells were induced by Si20nm only (**Fig. S12**). Interestingly, CD276 was only expressed on the DP and on the CD80+ single positive cells **(Fig. S12**). Both NPs tended to weakly increase CD276 on the activated DP cells at 24 hr. In summary, this data shows that DNFB and mTiO_2_ exposure promotes the upregulation of the activated DP phenotype, and increased PD-L1 expression in the naïve DN population over time. PD-L1 expression of the activated DP population was unchanged over time with exposure to DNFB and mTiO_2_, whereas Si20nm exposure tended to decrease PD-L1 expression pointing again to the similarities between DNFB and mTiO_2_.

### Effects of DNFB co-exposure with NPs on cytotoxicity, cytokine secretion and and co-stimulatory molecule expression

**Figure 5:**
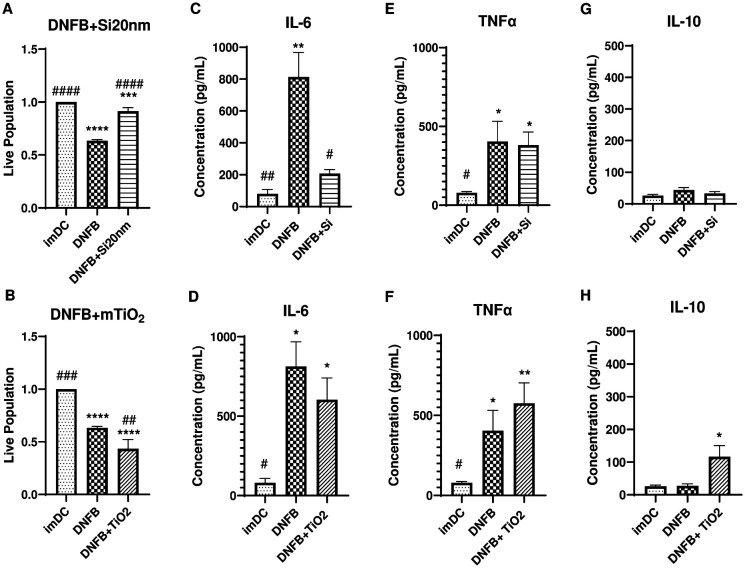
BMDC cytotoxicity and cytokine secretion following co-exposure to DNFB with Si20nm or mTiO_2_ NPs. BMDCs were co-exposed to DNFB plus Si20nm or mTiO_2_ nanoparticles for 24 hr. Cell viability and secreted cytokines were measured and compared to DNFB treatment alone and to untreated imDC as a control. (**A**) Si20nm NPs were shown to have cytoprotective effect in DNFB co-exposure whereas (**B**) mTiO_2_ NPs induced higher cytotoxicity. Supernatants were collected to analyze cytokine production by ELISA for (**C-D**) IL-6 which showed co-culture with Si20nm NPs decreased IL-6 production while co-culture with mTiO_2_ did not. (**E-F**) addition of either NPs did not alter the levels of TNFα produced by DNFB alone and (**G-H**) the co-culture of DNFB with mTiO_2_ NPs increased IL-10 production while Si20nm NPs did not. Ordinary one-way ANOVA was performed and compared to imDC (*) and DNFB alone (#). Concentration was normalized against % of live cells. N=3–5. Mean ± SD. */#p < 0.05, **/##p < 0.01, ***/###p < 0.001, ****/####p < 0.0001.

In prior studies NPs co-exposed with DNFB modulated the *in vivo* CHS response in the challenge phase^21^. In this work, we were able to observe clear differences between Si20nm and mTiO_2_ in single exposure studies of cytotoxicity, cytokine production, and on phenotypic alterations of BMDC, with mTiO_2_ behaving more similarly to DNFB. Here, we studied the effects of DNFB co-exposure with NPs on cytotoxicity and cytokine secretion compared to DNFB alone at 1 hr (**Fig. S13** and 24 hr (Fig. 5). Consistent with earlier studies (Fig. 1), BMDC cultured with DNFB (0.001 mM) alone for 24 hr was cytotoxic. Co-culture with Si20nm (0.01 mg/ml) showed a protective effect (Fig. 5A). In contrast, co-culture with mTiO_2_ (0.005 mg/ml) significantly exacerbated the toxic response (Fig. 5B). A cytoprotective effect of Si20nm was also observed at 1 hr exposure **(Fig. S13**) and is consistent with prior studies in keratinocytes^23^ and fibroblasts^61^. We also tested the supernatant of each treatment group for IL-6, TNFα, and IL-10 by ELISA. Results show a downregulation of IL-6 with DNFB co-cultured with Si20nm but not with mTiO_2_ (Figs. 5C–D). Co-culture with either NP did not alter the levels of TNFα produced by DNFB exposure at 24 hr (Figs. 5E–F) however, co-culture with mTiO_2_ showed elevated IL-6 levels at 1 hr and both NPs elevated TNFα above that produced by DNFB at 1 hr **(Fig. S13 E-F**). Co-culture with mTiO_2_, but not Si20nm (Figs. 5G–H), increased the secretion of IL-10, which is consistent with the mTiO_2_ single exposure studies (Fig. 2). These co-culture studies demonstrate that Si20nm exhibits a cytoprotective against effect DNFB exposure and exhibits an immunosuppressive effect as measured by a reduction in IL-6 secretion.

**Figure 6:**
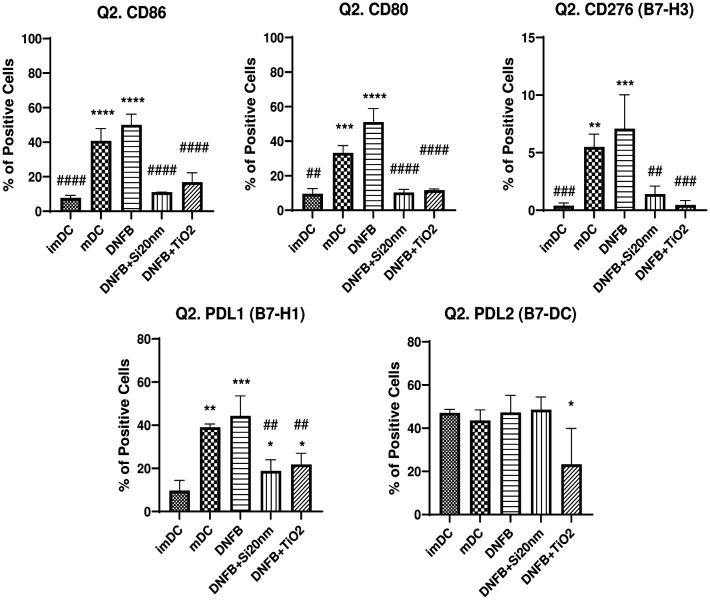
Co-stimulatory molecules from the B7 family quantified by flow cytometry gated on the CD11c+MHCII+ subpopulation following co-exposure of DNFB with NPs. BMDCs were treated with DNFB (0.001 mM DNFB) and co-exposed with Si20nm (0.01 mg/mL) or mTiO_2_ (0.005 mg/mL) NPs for 24 hr. Surprisingly, the addition of NPs appeared to suppressed activation of DNFB of all co-stimulatory molecules at 24 hr, except for PD-L2, for which mTiO_2_ significantly down regulated it. Ordinary one-way ANOVA was performed and compared to imDC (*) and DNFB (#). N=3–5. Mean ± SD.*/#p < 0.05, **/##p < 0.01, ***/###p < 0.001, ****/####p < 0.0001.

Next, the effects of DNFB co-exposure with NPs on the expression of the costimulatory molecules was investigated using flow cytometry (Fig. 6). Double gating on the CD11c+MHCII+ subpopulation unexpectedly showed that co-exposing BMDC to DNFB (0.001 mM) with either Si20nm (0.01 mg/ml) or mTiO_2_ (0.005 mg/ml) similarly down-regulated the DNFB activation of CD86, CD80, CD276, and PD-L1. Interestingly, co-exposure with mTiO_2_ downregulated PD-L2 expression to levels below the imDC levels.

**Figure 7:**
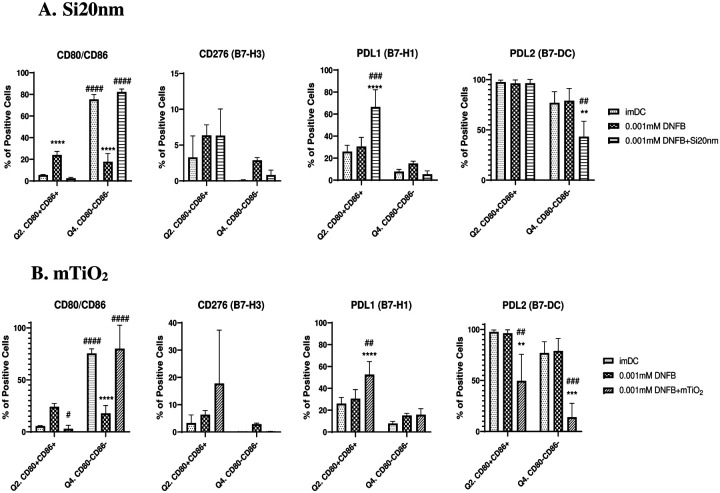
Co-stimulatory molecules from the B7 family quantified by flow cytometry gated on the CD11c+MHCII+ CD80/CD86 subpopulations following 24 hr DNFB alone or DNFB plus NP co-exposure. CD80/CD86 subpopulations were divided and CD276 (B7-H3), PD-L1 (B7-H1) and PD-L2 (B7-DC) were gated under, double positive (DP) population (Q2.) or double negative (DN) population (Q4.) in order to determine whether their expression varies under the different activation states of BMDCs by using the activation markers CD80/CD86 following non-cytotoxic concentrations of **(A)** 0.001 mM DNFB alone and plus 0.01 mg/mL Si20nm co-exposure and **(B)** 0.001 mM DNFB alone and pls 0.005 mg/mL mTiO_2_ co-exposure. NPs suppressed BMDCs activation, increased PD-L1 expression and mTiO_2_ induced a significant decrease in PD-L2 expression in the DP subpopulation at 24 hr while Si20nm did not. A two-way ANOVA was performed and compared to imDC (*) and DNFB (#). N=3–5. Mean ± SD. **/##p < 0.01, ***/###p < 0.001, ****/####p < 0.0001.

Differentiating the effects of DNFB co-exposure with NPs on the expression levels of PD-L1, PD-L2 and CD276 in the BMDC CD80/CD86 subpopulations was similarly less clear (Fig. 7) Consistent with single exposure studies at 24 hr (Fig. 4), DNFB activates the BMDCs as evidenced by a sharp increase in the C86+CD80+ DP subpopulation and a sharp decrease the naïve C86-CD80- DN subpopulation. Surprisingly, both NPs suppressed BMDC activation, maintaining the DP and DN subpopulations to imDC levels at 24 hr. There were no statistically significant changes in CD276 expression. However, both NPs co-cultured with DNFB increased PD-L1 expression on the DP subpopulation at 24 hr which was also evident at 1hr for mTiO_2_ but not for Si20nm, which exhibited a statistically significant decrease PD-L1 at 1 hr relative to the imDC (**Fig. S14)**. The most intriguing differential effect between the NPs co-cultured with DNFB was the observation that mTiO_2_ induced a significant decrease in PD-L2 expression in the DP subpopulation at 24 hr and Si20nm did not (Fig. 7). This decrease was evident after only 1 hr of co-culture (**Fig. S14)**. A second, potentially important difference between the NPs was observed at 1 hr exposure in PD-L1 expression on the DP subpopulation where Si20nm decreased expression whereas mTiO_2_ strongly upregulated it over DNFB levels **(Fig. S14)**. However, at 24 hr both NPs increased PD-L1 on the activated DP cells and decreased PD-L2 expression on the naive DN cells. It is interesting to note that statistically significant changes in PD-L2 expression were not observed in the activated DP or the naive DN subpopulations in single-exposure studies (Fig. 4). The significant differences in PD-L1 and PD-L2 expression in the activated DP subpopulation caused by mTiO_2_ co-cultured with DNFB and the opposing effects with Si20nm co-culture maybe influential in the BMDC controlling the fate of the adaptive immune response.

### Effects of NPs on the sensitization and challenge phases using the *in vivo* DNFB contact hypersensitivity model

**Fig. 8:**
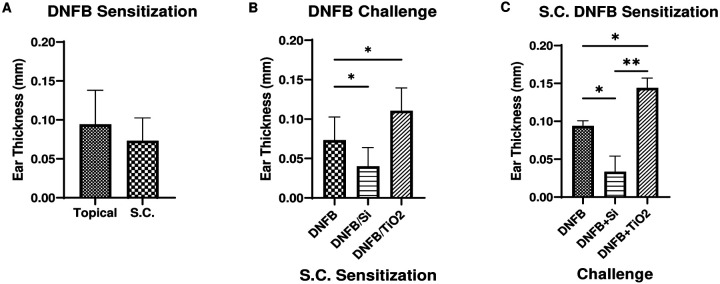
Comparison of CHS allergic response for different models of sensitization and challenge. (**A**) Comparison of ear swelling response for DNFB topical (0.05 %) vs. S.C. (0.01 mM DNFB) sensitization with 0.2% DNFB challenge on one ear vs. vehicle on the other. No differences between these sensitization methods was observed. (**B**) Comparison of ear swelling response for S.C. sensitization with BMDC treated 1 hr with DNFB only or DNFB+Si20nm NPs or DNFB+mTiO_2_ and challenge with 0.2% DNFB on one ear vs. vehicle on the other. Ear swelling was exacerbated with mTiO_2_ but decreased with Si20nm compared to DNFB alone. **(C)** Comparison of ear swelling response for S.C. sensitization with DNFB only and challenge with DNFB or DNFB co-exposure with NPs and vehicle on the other. Ear swelling was exacerbated with mTiO_2_ but decreased with Si20nm compared to DNFB alone. Ordinary one-way ANOVA was performed and compared to imDC (*). N=3–12. Mean ± SD. *p < 0.05, **p < 0.01.

Analysis of cytotoxicity, cytokine secretion and co-stimulatory markers taken together suggest that DNFB+Si20nm co-exposure tends to promote a more naïve or regulatory BMDC phenotype, whereas DNFB+mTiO_2_ promoted a proinflammatory BMDC phenotype. To test this, we used the *in vivo* CHS mouse model with adoptive transfer. We first tested the difference between topical and subcutaneous (S.C.) DNFB sensitization. BMDCs were treated with DNFB (0.01 mM) for 1 hr. After two wash steps, the cells in sterile saline were injected S.C. to sensitize the mice. A second group of mice was topically sensitized by applying 20 μl of 0.05 % DNFB in acetone:olive oil vehicle (4:1)^7,21,60^. Five days later, ear thickness was measured, and the mice were challenged with 0.2% DNFB on one ear and the vehicle on the other. On day 6 ear thicknesses were remeasured. Results of ear swelling response showed no difference between the topical sensitization and S.C. sensitization (Fig. 8A).

In prior work we observed an immunomodulatory effect of NPs in the challenge phase with DNFB topical sensitization^21^. To test if these effects are similarly observed with S.C. sensitization, we were able to sensitize (S.C.) the mice with BMDC treated with 0.01 mM DNFB for 1 hr. We challenged the mice on Day 5 with 0.2 % DNFB alone, or mixed with NPs and measured the ear thickness on Day 6. Results show a similar finding to our previous data^21^ indicating that Si20nm suppressed and mTiO_2_ exacerbated the allergic response relative to challenge with DNFB alone (Fig. 8B). Next, we compared S.C. sensitization using BMDC treated with DNFB (0.01 mM) alone or BMDC co-cultured with Si20nm (0.01 mg/ml) or mTiO_2_ (0.05 mg/ml) for 1 hr. After two wash steps, we S.C. injected the cells to sensitize the mice. Upon challenge, we treated one ear with 0.2 % DNFB and the other with vehicle. Results show that mice sensitized with BMDC treated with DNFB+Si20nm measured a decreased ear swelling relative to DNFB alone. In contrast, mice S.C. sensitized with BMDC treated with DNFB+mTiO_2_ showed an increase in ear swelling relative to DNFB alone (Fig. 8C). These results suggest that NPs co-cultured with a potent sensitizer can alter the BMDC phenotype to effect the efficiency of T cell priming and the intensity of the CHS reaction. In prior work we did not measure an effect of NPs co-exposed topically with DNFB in the sensitization phase^21^. This is most likely due to an inability of the NPs to breach the skin barrier to an appreciable extent to interact sufficiently with skin dendritic cells to alter their phenotype.

## DISCUSSION

Engineered NPs have broad applications in many industries and are extensively under development for biomedical use^62–64^. For example, NPs are being engineered for use in vaccine development where they act as adjuvants and/or carriers to generate antigen-specific tolerogenic adaptive immunity^64^. This is a superior therapeutic strategy compared to suppressing the entire immune system which, can cause long-term damage^65^. Hence, at the forefront of the nanomedicine and nanotoxicology fields, is the need to understand and control how NPs interact with the immune system ^66^.

This work expands on our previous studies that showed NPs can modulate the adaptive immune response in a CHS mouse model^21^. We showed that Si20nm NPs suppressed the allergic response in the challenge phase and mTiO_2_ exacerbated it^21–23^. The mechanism of how these NPs can alter adaptive immune responses remains unclear, which motivated this investigation to examine how these NPs could impact dendritic cell phenotype and function by quantifying BMDC cytotoxicity, cytokine production, the expression of the B7 family co-stimulatory ligands, and the *in vivo* adoptive transfer CHS model.

Results of this study show that BMDC treated with DNFB or mTiO_2_, as a function of time and increasing concentration, are cytotoxic (Fig. 1) and they produce higher levels of proinflammatory cytokines (Fig. 2) compared to Si20nm which exhibited a cytoprotective effect in DNFB co-culture studies whereas mTiO_2_ exacerbated DNFB cytotoxicity (Fig. 5). TEM studies confirm that both NPs were taken up by the BMDCs and it showed that DNFB exposure caused BMDCs to lose dendrites and increase the presence of lysosomes (**Fig. S2)**, which degrade exogenous materials^67^. mTiO_2_ exposure induced a significant presence of lipid droplets whereas Si20nm exposure showed only a small increase (**Fig. S2)**. An increase in lipid droplets may result from oxidative stress^68^, or it may indicate upregulation in metabolic activity through glycolysis which also drives the secretion of inflammatory cytokines^69^.

Analysis of the B7 family of co-stimulatory ligands suggest that DNFB and mTiO_2_ induce a proinflammatory BMDC (CD11c, MHCII+) phenotype by up-regulating CD86, CD80 and PD-L1 (Fig. 3) similar to LPS **(Fig. S10)** whereas, Si20nm had little effect and in fact promoted a more naïve phenotype by inducing a decrease in the percent of CD86+CD80+ cells over time (Fig. 4). Dendritic cells expressing low levels of CD80/CD86 present antigen poorly and may induce tolerance ^47,48^. It is interesting that potent proinflammatory stressors (LPS, DNFB, mTiO_2_) sharply upregulate the immunosuppressive PD-L1 ligand (Fig. 3, **Fig. S11)** which is a mechanism by which the PD-1/PD-L1 pathway balances the pro-inflammatory effect by promoting the development of Foxp3+ Tregs to limit the immune responses^43,45,70,71^.

Interestingly, the co-stimulatory molecule CD276 was found to be prominently expressed only on CD80+ cells (Fig. 4, **Fig. S12)** which, binds the inhibitory CTLA-4 receptor with high affinity^49,50^. Despite its link to promoting an immunosuppressive BMDC phenotype following activation of the arylhydrocarbon receptor ^60^, CD276 expression was down regulated by both NPs in our DNFB co-culture studies (Fig. 6), suggesting that CD276 plays a minimal role driving the adaptive immune response in this CHS adoptive transfer model. Unexpectedly, both NPs induced similar effects in modulating the expression levels of CD86, CD80, PD-L1 and CD276 induced by DNFB with the key exception of PD-L2, where mTiO_2_ downregulated PD-L2 on the activated CD86+CD80+ subpopulation and Si20nm did not (Figs. 6 and 7). This suggests an important role of PD-L2 in directing the efficiency T cell priming in the DNFB-CHS model where Si20nm treated BMDC suppresses the allergic response in both the sensitization phases whereas, mTiO_2_ exacerbated it (Fig. 8). This finding is consistent with studies of allergic asthma, that showed PD-L2 expression in the lung was protective against the initiation and progression of airway inflammation^72–75^. Our results point to the importance of PD-L2 expression in the sensitization phase with DNFB, a T 1 skewing hapten^76,77^ H. Studies suggest that PD-L1 and PD-L2 participate in the differential regulation of Th1 and Th2 cells.^78^ The PD-L1/PD-1 interaction causes a T_H_2 response and an increased IL-4 secretion while the PD-L2/PD-1 interaction causes a T_H_1 response and an increase in INF-γ secretion ^73^. In future studies it would be important to analyze full cytokine panels that contain T cell polarizing signals as well as chemokines important for lymph node trafficking. Quantifying the expression of the chemokine receptor CCR7 on the engineered BMDC is also important as it necessary for directing lymph node migration^79^. The percent of the S.C. injected BMDC that traffic to the lymph node could be determined using fluorescently labeled BMDC and correlating to CCR7 expression. Quantifying these metrics will be important for engineering tolerogenic dendritic cells is a promising therapeutic approach for treating autoimmune disorders and severe allergic disorders^65,80^.

While our studies point to differences in PD-L2 expression as a potential mechanism for the differential effects of Si20nm and mTiO_2_ on DNFB sensitization (Fig. 8), it is important to note that the adoptive transfer of *ex vivo* engineered BMDC preparations contains a heterogeneous mix of B7 phenotypes. Additional studies would be informative to examine the relative importance of each BMDC subset more fully in driving the allergic response. Specifically, the different CD86/CD80 subpopulations (DP, DN, single positive) could be sorted to test which phenotype induces potent allergic responses. Studies show that dendritic cells expressing high levels of CD80 but not CD86 are protective and can induce immune tolerance via promoting CD25+ regulatory T cells^81^. It is also important to investigate the effect of protein coronas that form on NPs exposed to biological fluids and cell culture media^82,83^. Differences in the corona protein composition or abundance could alter the NPs interaction with the BMDCs. Corona composition is highly dependent on surface charge^84^. Si20nm and mTiO_2_ are both negatively charged so we anticipate that similar compositional coronas would form but this should be confirmed in proteomics studies. It is also possible that culturing BMDC with DNFB may haptenize the cells making them directly antigenic. Injection of haptenized BMDC could activate endogenous antigen presenting cells (APCs) in the skin or in the lymph node. Sensitization via this mechanism could be confirmed using transgenic mouse models with deleted endogenous APCs, however, the striking differential effects of the NPs observed in sensitization phase suggest a role for the *ex vivo* engineered BMDC in directing the adaptive immune responses.

This study corroborated our earlier studies using topical DNFB sensitization that showed (Fig. 8B) these NPs affected the allergic response in the challenge phase with mTiO_2_ exacerbating the ear swelling and Si20nm suppressing it^21^. While the mechanism in the challenge phase remains unclear, it seems plausible that the NPs could modulate epidermal-derived signals that affect MC and/or DC activation. These signals could be alarmins produced by keratinocytes or even modulation of the endogenous expression of CD80 or CD86 on keratinocytes. Studies using transgenic mouse models that over expressed CD80 or CD86 on basal keratinocytes showed a differential ability induce a chronic inflammatory response in the DNFB CHS model, with CD80 overexpression inducing a prolonged ear swelling response with enhanced leukocyte infiltration^85^. The immunomodulation IL-10 cytokine was also persistently increased in the mouse ear skin in the CD80 transgenic mouse which is consistent with a potent proinflammatory response observed with LPS and mTiO_2_ treatment in this study.

In summary, this work points to metrics that can be used to predict the effects of NPs on contact allergy and points to the novel use of NPs to engineer immunomodulatory responses in contact allergy. Given that skin contact allergy is on the rise^1,2^, as is the creation of novel engineered nanomaterials for industrial, biomedical and consumer use^62–64,86^, there is a need for assays that can predict the impact that NPs may have on the immune response in the context of skin allergic disease.

Further, immunoengineering is an important growing field for developing cell-based therapies to induce antigen specific immune tolerance ^42,64,87,88^.

## MATERIALS AND METHODS

### Animals

Hairless SKH mice back-crossed 6 generations with C57BL/6 mice were used in this study. All animal experimental protocols were reviewed and approved by the University of Rochester Committee on Animal Resources (UCAR #2010–24E). Experiments involving animals and reporting of data were carried out in compliance with the ARRIVE guidelines and all methods were carried out in accordance with relevant guidelines and regulations.

### Cell Culture

BMDCs were harvested and cultured from tibiae and femurs of 8 to 12 weeks mice using a standard protocols^89^. Bone marrow cells were suspended in: RMPI 1640 (Gibco Cat# 11875–093) containing 10 % FBS (Gibco Cat# 10082147), 1 % pen strep (Gibco Cat# 15140–122), 2 mM Glutamax (Gibco Cat# 35050–061), 1 mM sodium pyruvate (Gibco Cat# 11360–070), 50 uM β-ME (Gibco Cat# 31350–010), and 20 ng/mL GM-CSF (Gibco Cat# PMC2011). A flow cytometry panel was defined (**Table. S1**) to characterize the development of BMDCs over time. While GM-CSF-derived BMDC cultures are heterogeneous, studies confirm they are comprised conventional DCs^90^ and BMDCs are extensively used in both fundamental research and in clinical protocols^91^. After 8 days of culture the majority of cells were CD11c+ (86.1 %), CD11b+ (98.1 %) and MHCII+ (67.6 %) with no detection of T or B cells (**Fig. S15**). On average 10 % of cells stain positive for F4/80 a macrophage marker and 71.3% of the cells were CD11c+, CD11b+ and MHCII+ triple positive. Eight-day old BMDCs were used for initiating all experiments.

### Silica and mTiO_2_ NP characterization

In prior studies we fully characterized the Si20nm (nanoComposix Cat# SISN20–25M) and mTiO_2_ (Sigma-Aldrich Cat# 677469) NPs using dynamic light scattering and zeta potential measurements ^21–23^. Transmission electron microscope (TEM) was used in this study to measure the size of the NPs, where the mTiO_2_ NPs were found to be 51.6 ± 12.5 nm and more polydisperse than Si20nm NPs which were found to be 20.6 ± 3.5 nm and more homogenous in size **(Figs. S3, S4).** These results align with prior studies showing that titanium dioxide has a greater tendency to agglomerate ^22^.

### Generation of BMDCs exposed to DNFB, Silica, and mTiO_2_ NPs

The BMDCs were exposed to DNFB, Si20nm, or mTiO_2_ at different concentrations for 1 hr and non-cytotoxic concentrations (0.001 mM DNFB, 0.01 mg/mL Si20 nm and 0.005 mg/mL mTiO_2_) were chosen for the 24 hr time studies and subsequent single exposure experiments. The co-exposure of DNFB with Si20nm NPs did not give any discernible toxicity *in vitro*, for which, the concentration of DNFB was increased ten-fold (0.01 mM) for some experiments. The concentrations for the co-exposure of DNFB with mTiO_2_ NPs remained at 0.001 mM and 0.005 mg/ml, respectively.

### Flow cytometry

For cell staining and analysis of co-stimulatory molecule expression, the antibodies and the concentrations used per 1M cells are summarized in **Table S1.** We used flow cytometry (Cytek Aurora, Cytek Biosciences) and FlowJo (v10.7.2) to analyze cells. The gating strategy is shown in **Fig. S9**.

### Cytotoxicity and Cytokine analysis

We normalized the viability of BMDCs by flow cytometry and live population against imDC that were not treated with any stressor and served as control. We collected the supernatant and analyzed the pro-inflammatory cytokines IL-6, TNFα and the immunosuppressive cytokine IL-10 by ELISA (Invitrogen Cat# 88–7064, 88–7324 and 88–7105, respectively) following manufacture instructions.

### Contact hypersensitivity (CHS) mouse model

Mice were sensitized topically on the back by applying 20 μl DNFB (0.05 %) diluted in an acetone and olive oil vehicle in a 4:1 ratio. After 5 days, we performed challenge with 20 μl of 0.2 % DNFB on one ear and vehicle (4:1 acetone and olive oil ratio) on the other ear. Alternatively, we sensitized the mice subcutaneously (S.C) by injecting BMDCs (2×10^6^) treated for 1 hr with DNFB (0.01 mM) only or DNFB (0.01 mM) plus NPs; Si20nm (0.01 mg/mL) or mTiO_2_ (0.005 mg/ml). After 5 days, we performed challenge with 20μl of 0.2 % DNFB on one ear, and the other ear treated with vehicle (4:1 acetone and olive oil ratio) or 0.2 % DNFB plus Si20nm (0.01 mg/ml) or mTiO_2_ (0.005 mg/ml) NPs. The magnitude of the allergic response post challenge was quantified by measuring ear thickness using a digital caliper (Mitutoyo Cat# 209–931) with a resolution of 0.005 mm on day 5, before the application of the challenge dose (pre-challenge). On day 6, both ears were remeasured (post-challenge). Ear swelling (mm) was measured as: (post-challenge) – (pre-challenge).

### Statistics

We used GraphPad Prism 9 to analyze all statistical analyses. Ordinary one-way or two-way analysis of variance (ANOVA) was used to compare expression levels of co-stimulatory molecules to imDC (*) and DNFB (#). imDC were not treated with any stressor and served as control. Two-tailed, unpaired with unequal variances, student’s t-test was used to compare the ear thickness between two different sensitizing treatments in the CHS *in vivo* study. P-values <0.05 were considered significant. All data are presented with standard deviation. The experiments were replicated at least three times.

## Data Availability

All data are available in the main text or the supplementary materials. Raw data will be made available upon request. Contact: Lisa DeLouise: lisa_delouise@urmc.rochester.edu
